# Potential Regulatory Role of miR-15b, miR-99b, and miR-181a of the Shikonin-Induced MAPK/ERK Apoptotic Signaling Pathway in Renal Carcinoma

**DOI:** 10.3390/biomedicines13122898

**Published:** 2025-11-27

**Authors:** Anna Vass, József Király, Erzsébet Szabó, Nitya Shree, Deisy Ramos, Mahua Choudhury, Petra Fodor, Krisztián Szegedi, Gábor Halmos, Zsuzsanna Szabó

**Affiliations:** 1 Department of Biopharmacy, Faculty of Pharmacy, University of Debrecen, 4032 Debrecen, Hungary; vass.anna@pharm.unideb.hu (A.V.); kiraly.jozsef@pharm.unideb.hu (J.K.); fodor.petra@pharm.unideb.hu (P.F.); 2 Doctoral School of Pharmaceutical Sciences, University of Debrecen, 4032 Debrecen, Hungary; 3 Department of Pharmacology, Faculty of Pharmacy, University of Debrecen, 4032 Debrecen, Hungary; erzsebet.szabo@med.unideb.hu; 4 HUN-REN-DE Pharmamodul Research Group, University of Debrecen, 4032 Debrecen, Hungary; 5 Department of Pharmaceutical Sciences, Irma Lerma Rangel School of Pharmacy, Texas A&M Health Science Center, College Station, TX 77845, USA; shree@tamu.edu (N.S.); deisy.ramos@tamu.edu (D.R.); mchoudhury@tamu.edu (M.C.); 6 Department of Urology, Faculty of Medicine, University of Debrecen, 4032 Debrecen, Hungary; szegedi.krisztian@med.unideb.hu

**Keywords:** shikonin, renal cell carcinoma, CAKI-2, A-498, miR-15b, miR-99b, miR-181a, specific target, apoptosis

## Abstract

**Background:** Shikonin, a natural compound derived from *Lithospermum erythrorhizon*, exhibits anticancer properties by inducing apoptosis in various tumor types, including clear cell renal cell carcinoma (ccRCC) cell lines CAKI-2 and A-498. This study investigates the mechanisms underlying shikonin-induced apoptosis, focusing on microRNAs miR-15b, miR-99b, and miR-181a in ccRCC. **Materials and Methods:** ccRCC cells were treated with 5 µM shikonin. Expression levels of miR-15b, miR-99b, and miR-181a were measured by TaqMan PCR. Apoptosis-related targets (AKT3, PDCD4, FOXO1, FOXO3, JNK1, and LAMTOR3) were identified in silico and validated by qRT-PCR and Western blot. Spearman’s correlation was used to evaluate miRNA–target relationships. Ingenuity Pathway Analysis explored relevant pathways. **Results:** Shikonin decreased miR-15b, miR-99b, and miR-181a levels in CAKI-2 cells, whereas these miRNAs were increased in A-498 cells, demonstrating cell-line-specific effects. qRT-PCR and Western blot confirmed changes in target expression, suggesting regulation by these miRNAs. In A-498 cells, miR-181a expression positively correlated with the studied target levels during 24–72 h of treatment, indicating that its potential regulatory role may be cell-type-dependent. MiR-15b and miR-99b showed linear correlations with targets in both cell lines, but expression patterns differed, suggesting direct regulation alongside potential involvement in additional pathways contributing to shikonin-induced apoptosis. **Conclusions:** Shikonin induces apoptosis in renal cancer cells by modulating the MAPK/ERK pathway and through cell-line-specific, cell-type-dependent regulation of miR-15b, miR-99b, and miR-181a. These findings highlight the importance of cell-type-dependent miRNA regulation and underscore the therapeutic potential of shikonin in ccRCC.

## 1. Introduction

Shikonin, a naturally occurring naphthoquinone pigment extracted from the root of *Lithospermum erythrorhizon*, has attracted considerable attention due to its multifaceted pharmacological properties. Traditionally used in Chinese medicine, shikonin exhibits diverse biological activities, including antimicrobial, anti-inflammatory, antithrombotic, antioxidant, wound-healing, and notably, antitumor effects [1,2]. Over the past two decades, increasing evidence highlights the cytotoxic effects of shikonin on various tumor cell types, including leukemia, hepatoma, melanoma, colorectal, and lung carcinoma cells [1]. These effects are primarily mediated through the induction of apoptosis, a regulated form of cell death critical for the removal of cancer cells [1,2,3].

Based on previous studies, shikonin induces apoptosis through the generation of reactive oxygen species (ROS), mitochondrial membrane disruption, and the activation of caspase cascades [4]. Importantly, shikonin also modulates several key intracellular signaling pathways that regulate cell death, including the PI3K/Akt and MAPK/ERK pathways [4,5]. The balance between cell proliferation and death is regulated through coordinated signal transduction pathways mediated by complex functional interactions between signaling axes. Among these, the PI3K/Akt, Ras/MAPK, and JAK/STAT pathways play dominant roles in promoting cell proliferation, differentiation, and survival [6].

MicroRNAs (miRNAs) are small, non-coding RNAs that regulate gene expression post-transcriptionally and are now recognized as central modulators of apoptosis, cell cycle, and tumor progression. Numerous miRNAs have been identified to influence apoptotic pathways [7]. For instance, miR-34a promotes apoptosis by downregulating anti-apoptotic proteins such as BCL-2 (BCL2 apoptosis regulator) and SIRT1 (sirtuin 1) [8]. Earlier studies have shown that miR-16 targets the anti-apoptotic proteins BCL-2 and MCL1 (MCL1 apoptosis regulator, BCL2 family member). Additionally, miR-29, miR-200c, and miR-125b have also been implicated in the regulation of apoptosis-related genes, including those involved in p53 signaling [9,10]. These findings collectively highlight the vital role of miRNAs in regulating apoptosis in tumor biology.

Among the miRNAs implicated in apoptosis, miR-15b, miR-99b, and miR-181a have gained attention in recent years. MiR-15b has been shown to target BCL-2 and influence the PI3K/Akt pathway, thus promoting apoptotic processes [11]. By targeting MAPK1 (mitogen-activated protein kinase 1), miR-15b-5p suppresses autophagy and promotes apoptosis in endothelial progenitor cells [12]. MiR-99b is known to downregulate mTOR (mammalian target of rapamycin), a crucial downstream effector of the PI3K/Akt pathway, which controls cell growth and survival [13].

MiR-181a has also been reported to modulate the MAPK/ERK signaling pathway and play a role in the fine-tuning of apoptosis-related transcription factors and kinases [14,15].

These miRNAs are frequently deregulated in various cancers, including renal cell carcinoma (RCC), and may serve as molecular switches that determine cellular sensitivity to apoptosis-inducing agents such as shikonin.

In our earlier study [11], multiple apoptotic protein analyses demonstrated that shikonin may exert its pro-apoptotic effects through key proteins involved in the Ras/MAPK and PI3K/Akt signaling pathways [11]. Notably, these effects appeared to occur independently of the direct epigenetic regulation by the investigated miRNAs, such as miR-21 and miR-155. However, the potential involvement of additional miRNAs in shikonin-induced apoptosis in renal cancer remains unclear and warrants further investigation through theoretical analysis and experimental validation. Based on our findings, we propose that additional in vivo studies are essential to evaluate the possible contribution of additional miRNAs to apoptotic mechanisms initiated by shikonin.

A separate study by Király et al. investigated the roles of miR-15b, miR-99b, and miR-181a in the regulation of angiogenesis [11,16]. Our previous work supports the hypothesis that these three miRNAs are key modulators of angiogenic pathways that may contribute to kidney tumor development. Notably, they are also involved in other crucial cellular processes, including apoptosis, autophagy, and cell migration [11,16].

Although miR-15b, miR-99b, and miR-181a are known to play important roles, their involvement in the apoptotic response to shikonin in RCC cells has not yet been clarified. Given that these miRNAs have emerged as promising therapeutic targets and diagnostic biomarkers in oncology, elucidating how shikonin influences specific miRNA networks may facilitate the development of novel miRNA-based anticancer strategies [16].

Various human renal cell carcinoma cell lines are frequently used in studies evaluating drug response and the molecular mechanisms of tumor progression. Results obtained from earlier studies can provide a more comprehensive understanding of renal cancer biology. CAKI-2 represents a well-differentiated ccRCC with epithelial characteristics, whereas A-498 displays a more aggressive phenotype and a distinct genetic background. Therefore, the use of CAKI-2 and A-498 cell lines derived from ccRCC represents a consistent and appropriate choice for in vitro investigations, as it enables meaningful comparison with data obtained from human kidney cancer tissues [17,18].

The main goal of this study was to examine the effects of shikonin on the expression of miR-15b, miR-99b, and miR-181a in CAKI-2 and A-498 cell lines. Considering their potential roles in regulating apoptosis through the PI3K/Akt and MAPK/ERK signaling pathways, we aimed to assess whether these miRNAs participate in shikonin-induced apoptotic processes. In addition, in silico analyses were performed to predict apoptosis-related target genes of these miRNAs. Subsequently, we analyzed the expression of these candidate target genes to clarify their contribution to the downstream effects of shikonin-mediated miRNA modulation. Overall, this study seeks to deepen our understanding of the molecular interactions underlying natural compound-mediated epigenetic regulation in renal cell carcinoma.

## 2. Materials and Methods

### 2.1. Cell Lines

Two distinct human clear cell renal cell carcinoma cell lines, A-498 and CAKI-2 from ATCC (Rockville, MD, USA), were used in our study. Iscove’s Modified Dulbecco’s Medium (IMDM-Biosera, Cholet, France) supplemented with antibiotics (100 U/mL penicillin and 100 µg/mL streptomycin) and 10% Fetal Bovine Serum (FBS-Biosera, Cholet, France) was used as culturing medium for both cell lines. Cells were cultured at 37 °C with 5% CO_2_ in a T75 flask and passaged twice a week for maintenance.

### 2.2. Shikonin Treatment of CAKI-2 and A-498 Cells

Shikonin was purchased from MedChemExpress and prepared according to the instructions of Santa Cruz Biotechnology (Dallas, TX, USA). The stock solution was prepared in DMSO according to the manufacturer’s instructions and stored at −20 °C in 10 µL aliquots. Two human ccRCC cell lines, A-498 and CAKI-2, were used for the experiments [16]. In our earlier study, it was published that shikonin significantly suppressed the cell proliferation of both A-498 and CAKI-2 cells in a time- and dose-dependent manner as early as after 24 h at a 2.5 µM concentration [11]. Based on our previous experimental results, we selected a higher concentration of 5 μM shikonin for further experiments, as this dose provided a strong biological response while remaining below cytotoxic levels. This 5 μM concentration was also used in our earlier studies with CAKI-2 and A-498 cells, ensuring consistency and comparability among experiments [11].

### 2.3. RNA Isolation from Cell Lines

CAKI-2 and A-498 human kidney cancer cells cultivated in T75 flasks with 80% confluency were lysed using TRIzol reagent (TR118, Molecular Research Center Inc, Cincinnati, OH, USA) followed by RNA extraction according to the Chomczynski and Sacchi method [19]. DNase I digestion was performed in all samples after RNA extraction and prior to cDNA synthesis. This step was included in the RNA isolation protocol (rDNase Set, Macherey–Nagel, Cat. No. 10845761, Düren, Germany). After determining the RNA solution concentrations and purity using NanoDrop 1000 spectrophotometer (Nanodrop Technologies, Wilmington, DE, USA), the samples were kept at −70 °C for future usage.

### 2.4. Reverse Transcription PCR (RT-PCR)

Complementary DNA (cDNA) was synthesized using the Tetro cDNA Synthesis Kit (BIO-65043, BIOLINE, London, UK) according to the manufacturer’s instructions. Reverse transcription was performed in a thermal cycler (C1000™ Thermal Cycler, Bio-Rad Laboratories Inc., Hercules, CA, USA). The reaction mixture was incubated at 45 °C for 30 min to allow RNA and primers to hybridize and enable cDNA synthesis. This was followed by heating at 85 °C for 5 min to inactivate the enzyme and denature RNA–cDNA hybrids. The mixture was then cooled on ice. The resulting cDNA was stored at −20 °C for further use.

### 2.5. TaqMan microRNA Assay

Quantitative real-time PCR (qRT-PCR) was performed using the TaqMan^®^ MicroRNA Assay system (Life Technologies, Carlsbad, CA, USA). Complementary DNA (cDNA) for each miRNA was synthesized from 5 ng of total RNA using TaqMan^®^ MicroRNA Reverse Transcription Reagents (Invitrogen, Carlsbad, CA, USA) together with miRNA-specific stem-loop primers (Life Technologies, Carlsbad, CA, USA). Real-time PCR amplification was conducted in a CFX-96 Real-Time PCR System (Bio-Rad Laboratories Inc., Hercules, CA, USA) under the following cycling parameters: an initial denaturation step at 95 °C for 10 min, followed by 40 cycles of 95 °C for 30 s and annealing at 60 °C for 1 min. All reactions were carried out in triplicate using 96-well plates.

Cycle threshold (Ct) values were automatically calculated with SDS software version 3.1 (Applied Biosystems, Foster City, CA, USA). RNU4B and RNU6B (U4 and U6 small nuclear RNAs) were used as endogenous references to normalize miRNA expression levels. The data was processed in the R statistical environment (v. 2.14.0, Applied Biosystems, Foster City, CA, USA), where triplicate C_T_ values were averaged and normalized to the geometric means of RNU4B and RNU6B. These reference genes were selected based on their expression stability, which was verified using the geNorm and NormFinder algorithms (Department of Molecular Medicine, Aarhus University Hospital, Aarhus, Denmark). Normalized expression values were calculated using the formula 2^−ΔΔCT^, and Ct values above 36 were considered below the detection threshold.

### 2.6. In Silico miRNA Analysis for Target and Pathway Prediction

A comprehensive in silico analysis was conducted to predict the target genes of microRNAs hsa-miR-15b-5p, hsa-miR-99b-3p, and hsa-miR-181a-5p. This computational approach was initiated based on existing literature that implicated these specific miRNAs in various cellular processes. The in silico analysis involved a detailed comparison of predicted miRNA-specific targets across three bioinformatics databases: miRDB, TargetScan, and TarBase. Predicted miRNA targets were defined as the intersection of miRDB and TargetScan, while all experimentally validated targets from TarBase were included. This approach integrates computational predictions with validated targets, ensuring that no experimentally confirmed genes are excluded.

In our earlier study, we also demonstrated that treatment with shikonin inhibited the phosphorylation of p44/42 MAPK (pErk) in A-498 and CAKI-2 kidney cancer cells, leading to apoptosis via a mitochondria-dependent pathway. Other studies have also reported similar effects of shikonin in various cancer cell types. These findings supported the anti-proliferative effect of shikonin [6,11,20,21,22]. Therefore, the primary objectives of this extensive in silico screening were to identify target proteins significantly involved in the apoptosis signaling pathway and elucidate miRNA-mediated regulation of this pathway.

Based on these in silico analyses for identification of apoptosis-related targets, predicted targets were retrieved from miRDB and TargetScan, and experimentally validated targets were obtained from TarBase. Only genes with documented apoptotic functions based on literature or database annotations were retained. The resulting target sets were further analyzed using Ingenuity Pathway Analysis (IPA, Qiagen) to confirm involvement in apoptosis signaling pathways.

### 2.7. Ingenuity Pathway Analysis

We performed an in-depth analysis of the molecules of our interest using Ingenuity Pathway Analysis (Qiagen, Redwood City, CA, USA). The relationship of shikonin with the disease and function linked to renal cancer and apoptosis was established in pathway analysis. We also visualized the direct and indirect relationships between the nodes (identified molecules) using Path Designer. Direct relationships are represented by a solid line, and indirect relationships are represented by a dashed line (Figure S1).

### 2.8. Quantitative Real-Time PCR (qRT-PCR) Analysis

Quantitative real-time polymerase chain reaction (qRT-PCR) was performed using the iTaq™ Universal SYBR^®^ Green Supermix (Bio-Rad Laboratories Inc., Hercules, CA, USA) to quantify gene expression. All reactions were carried out in a CFX-96 Real-Time System (Bio-Rad Laboratories Inc., Hercules, CA, USA) with a total reaction volume of 20 µL.

The thermal cycling protocol began with an initial denaturation step of 3 min at 95 °C, followed by 40 cycles of a two-step amplification: denaturation at 95 °C for 15 s and an annealing and extension phase at 60 °C for 1 min. The specific oligo sequences for each target gene are listed in Table S1. To ensure experimental and statistical reliability, all reactions were performed in triplicate.

Following amplification, the relative messenger RNA (mRNA) expression levels were calculated using the comparative threshold cycle method (ΔΔC_T_). GAPDH (glyceraldehyde 3-phosphate dehydrogenase) was selected as the endogenous control due to its stable expression across most experimental conditions, making it suitable for normalization. The relative fold change in gene expression was calculated using the 2^−ΔΔCT^ method, expressed as log2|2 − ΔC_T_|.

### 2.9. Protein Isolation and Western Blot

Human CAKI-2 and A-498 kidney cancer cells were exposed to 5 µM shikonin for 24, 48, and 72 h. Following treatment, cells were rinsed with PBS and lysed using M-PER protein lysis buffer supplemented with protease and phosphatase inhibitors. Protein concentrations in the lysates were determined using the Bradford assay. Each sample was combined with 4× Laemmli loading buffer and heated at 95 °C for 8 min to denature proteins. Proteins (40 μg per lane) were separated on SDS-PAGE (10% or 14%, depending on molecular weight standards) using the Precision Plus Protein Dual Color Standard (Bio-Rad Laboratories Inc., Hercules, CA, USA) as a molecular size marker. After the electrophoresis process was completed, proteins were transferred onto PVDF membranes. Membranes were incubated with primary antibodies (Table S2) diluted at 1:1000, then probed with horseradish peroxidase-linked secondary antibodies (1:2000) (Thermo Fisher Scientific, Waltham, MA, USA). Detection was performed using a chemiluminescent substrate. Bands were imaged on a ChemiDoc Touch system (Bio-Rad Laboratories, Hercules, CA, USA). To verify uniform loading, band intensities were normalized against Hypoxanthine Phosphoribosyltransferase (HPRT, Sigma-Aldrich, St. Louis, MO, USA) or Glyceraldehyde-3-phosphate dehydrogenase (GAPDH, Cell Signaling Technology, Danvers, MA, USA). Band intensities were quantified using the Bio-Rad Image Lab software v5.2.1 (Bio-Rad Laboratories, Hercules, CA, USA). The detected protein bands were aligned and adjusted for accurate lane matching. Following this, band intensity values were quantified based on the measured signal intensity of each protein and normalized to the corresponding housekeeping protein (HPRT or GAPDH). The normalized data were then exported to Excel for further analysis. All experiments were performed with three biological replicates.

### 2.10. Statistical Analysis

All experiments were conducted in three independent biological replicates (*n* = 3). For qRT-PCR, three technical replicates (*n* = 3) were performed for each biological replicate. Western blot analyses were repeated three times using independent biological replicates. Data processing, including calculation of mean values, standard deviations, and normalization as percentages, was carried out using Microsoft Excel (Office Professional Plus 2016, Microsoft, Redmond, WA, USA). Statistical analyses were performed with GraphPad Prism version 9.5.1 (GraphPad Software Inc., San Diego, CA, USA) using one-way ANOVA. A *p*-value less than 0.05 was considered statistically significant. Statistical significance was determined across all biological replicates. In all graphs, individual data points representing biological replicates are shown, with error bars indicating SEM.

### 2.11. Correlation Analysis of the Expression of miRNAs and Their Specific Targets

To explore the relationship between the expression of identified microRNAs and the mRNA levels of those common specific targets identified in target databases, correlation analysis was conducted. Spearman’s rank correlation, a nonparametric method, was used to determine correlation coefficients (r) between miRNA and mRNA expression levels. The reliability of each correlation was assessed using a 95% confidence interval (CI) for the r value. When a statistically significant correlation (*p* < 0.05) was identified, linear regression was performed and visualized with 95% confidence bands around the best-fit line.

## 3. Results

### 3.1. Shikonin Regulates microRNA Expression in CAKI-2 and A-498 Cell Lines Differently

**Figure 1:**
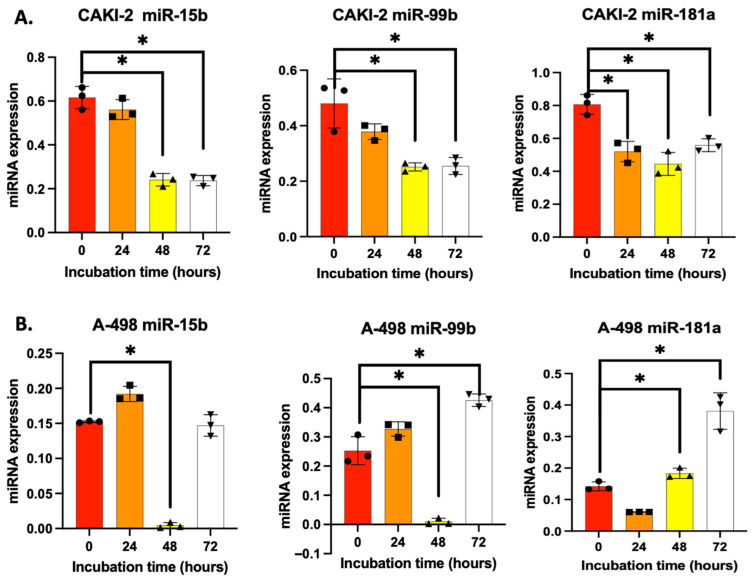
Effect of shikonin on the expression of miRNAs of interest (hsa-miR-15b-5p, hsa-miR-99b-3p, and hsa-miR-181a-5p) after 24, 48, and 72 h of treatment in CAKI-2 (**A**) and A-498 (**B**) cells. The geometric means of RNU4B and RNU6B, selected based on geNorm and NormFinder stability analyses, served as an endogenous control, allowing for normalization of each target miRNA. The presented data is based on three independent experiments (*n* = 3), each performed in triplicate (*n* = 3). One-way ANOVA with Sidak multiple comparison test was used for statistical analysis (* *p* < 0.05).

Shikonin treatment (5 μM) resulted in a decreased expression of miR-15b in CAKI-2 cells at 24, 48, and 72 h, respectively, compared to untreated control cells. In contrast, A-498 cells exhibited an initial increase in miR-15b expression at 24 h, followed by a sharp decline at 48 h, and a subsequent increase at 72 h (Figure 1A).

In CAKI-2 cells, miR-99b expression showed a continuous decline at all time points relative to the control group. However, in A-498 cells, miR-99b expression increased at 24 and 72 h following treatment (Figure 1B).

In CAKI-2 cells, miR-181a expression consistently decreased at all examined time points when compared to control cells. Conversely, in A-498 cells, miR-181a expression decreased at 24 h but increased after 48 and 72 h of shikonin treatment (Figure 1A,B).

### 3.2. In Silico Identification of Apoptosis-Related miRNAs Targets

To identify the potential target genes involved in apoptotic pathways, an in silico analysis was conducted using three different microRNA target prediction databases. The aim was to determine overlapping targets regulated by selected microRNAs. After compiling and numerically plotting the predicted targets from each database, we performed a comparative analysis to identify common genes among the datasets.

Only targets with documented roles in apoptosis were retained for further analysis, as confirmed through literature-based functional annotations and Ingenuity Pathway Analysis (IPA, Qiagen), ensuring that all identified miRNA–mRNA interactions were specifically associated with apoptotic signaling.

Using miRDB, TargetScan, and TarBase, AKT3 (serine/threonine kinase 3), MAPK3 (mitogen-activated kinase 3), BCL-2, and LAMTOR3 (late endosomal/lysosomal adaptor, MAPK and MTOR activator) were consistently identified as four common predicted targets of miR-15b (Table S3). This consistent identification across all three databases supports their potential regulatory roles in apoptotic signaling and survival pathways.

**Figure 2:**
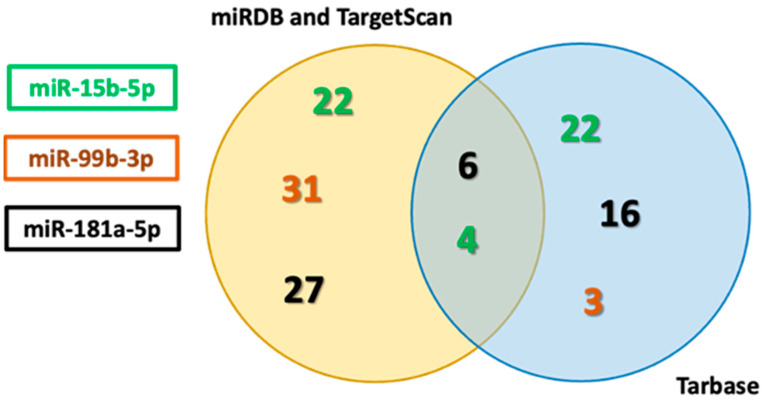
Comparison of predicted and experimentally validated miRNA target genes. The figure illustrates the overlap between the predicted and the validated miRNA targets obtained from three databases. Predicted targets were defined as the intersection between miRDB and TargetScan, and experimentally validated targets were obtained from TarBase. This separation highlights both the overlap and the complementarity between prediction-based and experimentally supported miRNA–mRNA interactions. Table S3 provides a detailed list of the targets that were consistently identified across all three databases, indicating a higher degree of confidence in their association with the miRNAs.

Predicted targets (shared between miRDB and TargetScan), along with experimentally validated targets from TarBase, are presented as separate data categories to illustrate both the overlap and the complementarity between computational predictions and validated results (Figure 2, Table S3).

For miR-99b, eight common predicted targets—DEDD (death effector domain-containing protein), NIBAN1 (niban apoptosis regulator 1), SOX4 (SRY-box transcription factor 4), JNK1 (c-Jun N-terminal kinase 1), TNFSF10 (TNF superfamily member 10), MAPK9, ERK (extracellular signal-regulated kinase) and FGF12 (fibroblast growth factor 12)—were identified using miRDB and TargetScan. These targets were not confirmed in TarBase, indicating they have not yet been experimentally validated or uploaded in that database. Within TarBase, BCL2L11 (Bcl-2-like protein 11) emerged as a specific, validated target related to apoptotic regulation (Table S3).

For miR-181a, six targets—AKT3, FOXO1 (forkhead box O1), FOXO3 (forkhead box O3), BCL-2, PDCD4 (programmed cell death 4), and LAMTOR3 (late endosomal/lysosomal adaptor)—were consistently identified across all three databases, supporting their potential regulatory role in apoptotic signaling and survival pathways (Table S3).

This analysis revealed a set of common targets, including several key regulators of apoptosis and cell survival: AKT3, PDCD4, FOXO1, FOXO3, JNK1, ERK and LAMTOR3 (Figure 2 and Table S3). The convergence of these targets across multiple prediction platforms highlights their potential biological relevance and suggests that they may represent critical nodes within the miRNA-mediated regulation of apoptosis.

### 3.3. Validation of Putative Target Gene Expression by qRT-PCR

The expression levels of the putative miRNA target genes were assessed using quantitative real-time PCR (qRT-PCR) with gene-specific primers (Table S1).

qRT-PCR analysis showed that AKT3 and PDCD4 expression levels were markedly upregulated in A-498 cells at 24, 48, and 72 h following shikonin treatment. In contrast, CAKI-2 cells displayed only a modest increase in the expression of these genes, observable primarily at 72 h post-treatment.

**Figure 3:**
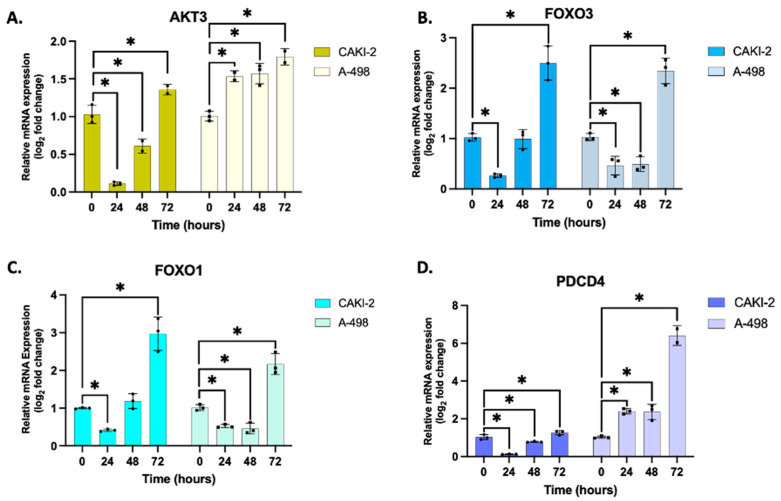
Effect of shikonin on the expression of (**A**) AKT3, (**B**) FOXO3, (**C**) FOXO1, and (**D**) PDCD4 genes. Following treatment with 5 µM shikonin, total RNA was extracted from all samples at 24, 48, and 72 h, using three biological replicates (*n* = 3), each performed in triplicate (*n* = 3). Gene expression profiling was conducted using 40 ng of cDNA per sample, with GAPDH as the housekeeping gene for normalization. Relative expression levels in shikonin-treated samples were normalized to their respective untreated controls using the 2^−ΔΔCT^ method. Data represents the mean log_2_ fold change ± standard error of the mean (SEM). One-way ANOVA with Sidak’s multiple comparison test was used to detect significant differences (* *p* < 0.05). Primer sequences are listed in Table S1.

In addition, the expression patterns of FOXO1 and FOXO3 were similar across both cell lines. Their expression levels increased significantly after 72 h of shikonin exposure, suggesting a delayed but coordinated activation of these transcription factors in response to treatment (Figure 3).

Fold changes relative to the control group, calculated from three technical replicates (*n* = 3) across three independent biological replicates (*n* = 3), show the differences in the expression of the examined target genes in both CAKI-2 and A-498 cell lines (Table S4).

**Figure 4:**
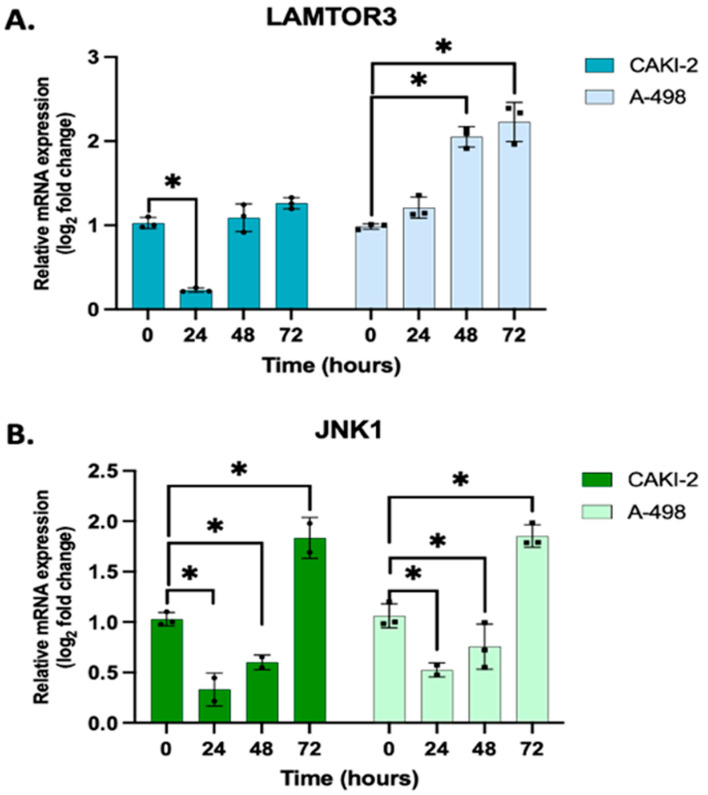
Effect of shikonin on the expression of (**A**) LAMTOR3 and (**B**) JNK1 genes. Following treatment with 5 µM shikonin, total RNA was extracted from all samples at 24, 48, and 72 h, using three biological replicates (*n* = 3), each performed in triplicate (*n* = 3). Gene expression profiling was conducted using 40 ng of cDNA per sample, with GAPDH as the housekeeping gene for normalization. Relative expression levels in shikonin-treated samples were normalized to their respective untreated controls using the 2^−ΔΔCT^ method. Data represents the mean log_2_ fold change ± standard error of the mean (SEM). One-way ANOVA with Sidak’s multiple comparison test was used to detect significant differences (* *p* < 0.05). Primer sequences are listed in Table S1.

Based on the real-time qPCR results, it is assumed that both the MAPK/ERK and JNK pathways might affect the development of kidney cells. After 72 h of shikonin treatment, the expression of genes JNK1 and LAMTOR3 evidently increased in both cell lines of the studied human kidney cancer (Figure 4).

### 3.4. Correlation of the Expression of the Studied miRNAs and Their Putative Target

**Figure 5:**
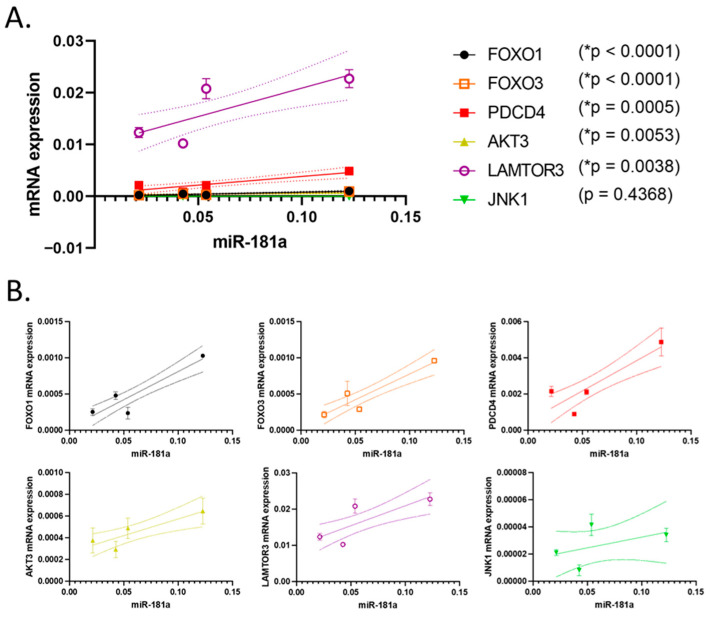
Correlation of the expression of miR-181a to the level of mRNA expression of targets FOXO1, FOXO3, PDCD4, AKT3, LAMTOR3 and JNK1 in A-498 cells. (**A**) A positive correlation was observed between miR-181a expression and the mRNA expression of FOXO1, FOXO3, PDCD4, AKT3 and LAMTOR3 (* *p* < 0.05); (**B**) shows values separately for better clarity. Data points represent the mean ± standard error (SEM) of three technical replicates (*n* = 3). Straight lines indicate the best-fit regression lines, and dotted lines represent the 95% confidence intervals derived from linear regression analysis.

The relative expression of miR-181a was analyzed in relation to the mRNA expression levels of the specific targets, such as FOXO1. FOXO3, PDCD4, AKT3, JNK1 and LAMTOR3. In the A-498 kidney cancer cell, a positive correlation (*p* < 0.05) was observed between miR-181a expression and the mRNA expression of FOXO1, FOXO3, PDCD4, AKT3, JNK1 and LAMTOR3 (Figure 5). Although linear correlations were observed for miR-15b and miR-99b, the two cell lines exhibited different expression patterns for both these miRNAs and the analyzed genes (Figure S2).

### 3.5. Shikonin Regulates the Expression of miRNAs’ Specific Target Proteins Related to Apoptosis

**Figure 6:**
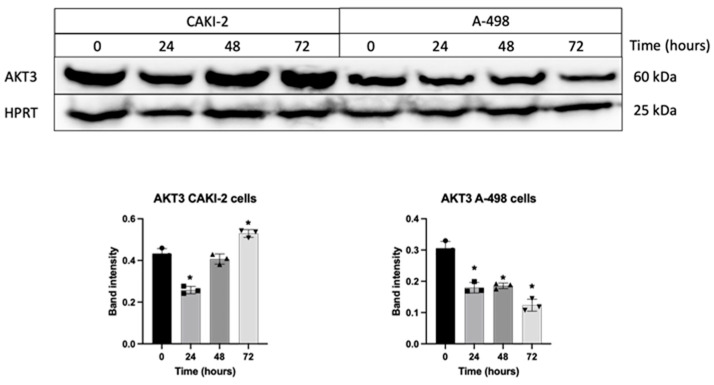
Detection of changes in the protein expression of AKT3 after treatment with shikonin for 24–72 h. Cells were exposed to 5 µM shikonin for 24, 48, and 72 h, followed by the isolation of total protein. Subsequently, 40 µg of protein from each sample was separated on a polyacrylamide gel, and the expression of AKT3 was determined. Quantification of protein band intensities was performed using Image Lab software (Bio-Rad Laboratories Inc.), and the intensity of shikonin-treated samples of AKT3 was normalized to HPRT levels. Data from three distinct experiments (*n* = 3) are presented as the mean ± standard error (SEM), and significant differences were evaluated using one-way ANOVA (* *p* < 0.05). (Page 1 in File S1. Original blot images).

According to our Western blot analyses, in CAKI-2 cell lines, AKT3 protein expression was found to be upregulated following shikonin treatment. In contrast, in A-498 cell lines, AKT3 protein levels steadily decreased compared to untreated control cells (Figure 6).

**Figure 7:**
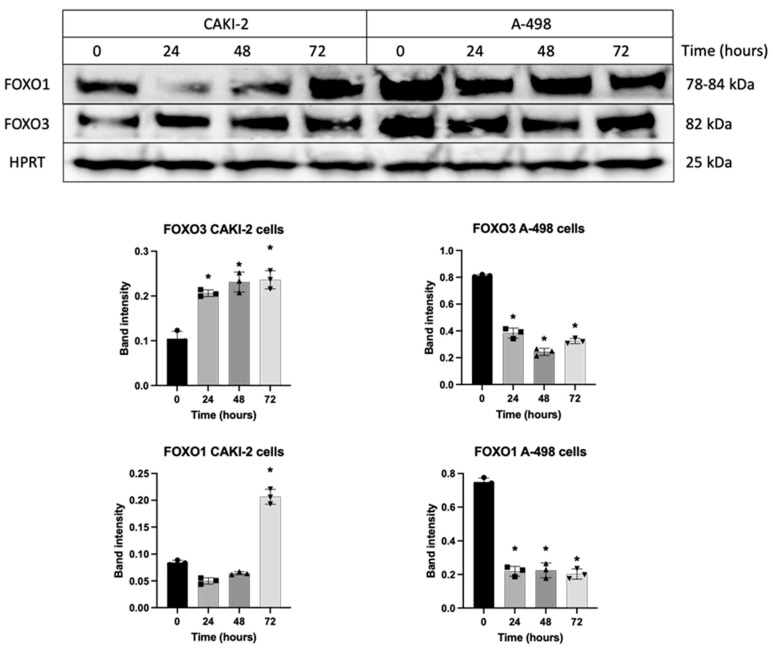
Changes in FOXO1 and FOXO3 protein expression were analyzed following treatment with shikonin for 24 to 72 h. Cells were exposed to 5 µM shikonin for 24, 48, and 72 h, after which total protein was extracted. A total of 40 µg of protein was loaded onto a polyacrylamide gel, and the expression levels of FOXO1 and FOXO3 were measured. Band intensities were quantified using Image Lab software (Bio-Rad Laboratories Inc.), and the shikonin-treated sample intensities were normalized against HPRT, a housekeeping protein. Data from three distinct experiments (*n* = 3) are presented as the mean ± standard error (SEM), and significant differences were evaluated using one-way ANOVA (* *p* < 0.05). (Pages 2 and 3 in File S1. Original blot images).

**Figure 8:**
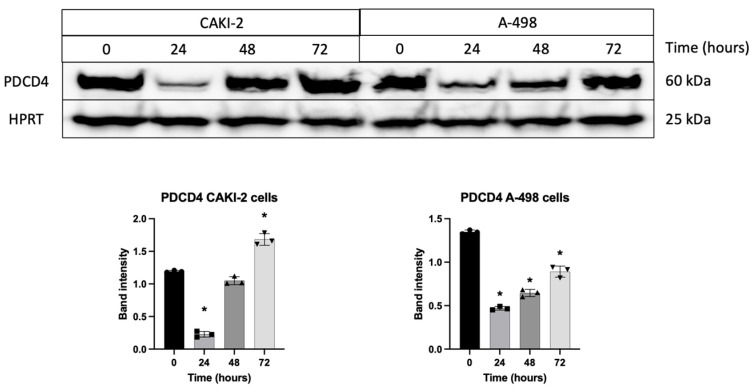
The expression of PDCD4 protein was measured after cells were treated with 5 µM shikonin for 24, 48, and 72 h. Total protein was extracted at each time point, and 40 µg of protein were separated using polyacrylamide gel electrophoresis. PDCD4 expression was evaluated, and band intensities were quantified using Image Lab software (Bio-Rad Laboratories Inc.). The intensity of the shikonin-treated samples was normalized to HPRT as the housekeeping protein. Data from three distinct experiments (*n* = 3) are presented as the mean ± standard error (SEM), and significant differences were evaluated using one-way ANOVA (* *p* < 0.05). (Page 4 in File S1. Original blot images).

Upon treatment with 5 µM shikonin for 72 h, CAKI-2 cells showed a significant increase in FOXO1, FOXO3 and PDCD4 protein expression (Figure 7 and Figure 8). However, PDCD4 expression initially declined at 24 h, followed by an increase at 72 h in these cells. In contrast, FOXO1, FOXO3 and PDCD4 protein levels in A-498 cells showed a decrease after 72 h of shikonin treatment (Figure 7 and Figure 8).

**Figure 9:**
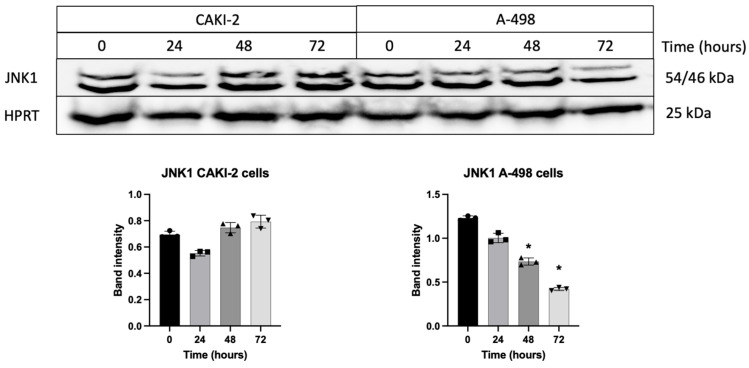
Following treatment with 5 µM shikonin for 24–72 h, changes in JNK1 protein expression were evaluated in CAKI-2 and A-498 cells. After each treatment interval (24, 48, and 72 h), total protein was extracted, and 40 µg of protein were subjected to SDS-PAGE. JNK1 expression was analyzed, and band intensities were quantified using Image Lab software (Bio-Rad Laboratories Inc.). Results were normalized to HPRT, used as a housekeeping control. Data from three distinct experiments (*n* = 3) are presented as the mean ± standard error (SEM), and significant differences were evaluated using one-way ANOVA (* *p* < 0.05). (Page 5 in File S1. Original blot images).

In the CAKI-2 cell line, JNK1 expression increased after 72 h of shikonin treatment compared to the control cells. In contrast, the A-498 cell line exhibited a gradual decrease during the 72 h treatment (Figure 9).

**Figure 10:**
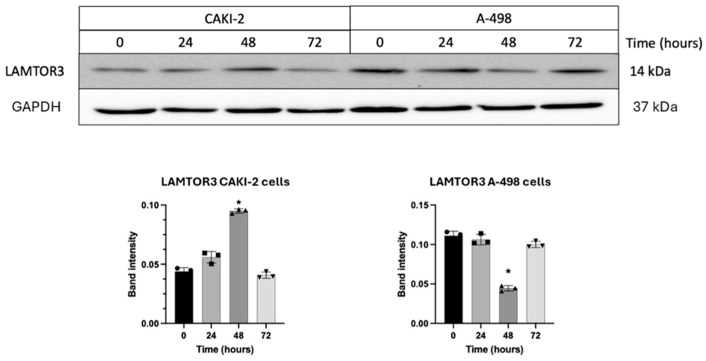
Changes in LAMTOR3 (MAPK2K1IP1) protein expression were evaluated following treatment with 5 µM shikonin. Cells were treated for 24, 48, and 72 h, after which total protein was isolated. 40 µg of protein were separated by polyacrylamide gel electrophoresis, and LAMTOR3 expression was analyzed. Band intensities were quantified using Image Lab software v5.2.1 (Bio-Rad Laboratories Inc.) and normalized to GAPDH, used as a housekeeping protein. Data from three distinct experiments (*n* = 3) are presented as the mean ± standard error (SEM), and significant differences were evaluated using one-way ANOVA (* *p* < 0.05). (Page 6 in File S1. Original blot images).

Western blot analyses revealed that shikonin treatment caused a transient elevation in LAMTOR3 protein levels in CAKI-2 cells, with a peak at 48 h post-treatment compared to the untreated control cells. This elevation was temporary, as expression levels returned to baseline by 72 h. In contrast, A-498 cells exhibited a higher basal expression of LAMTOR3 relative to CAKI-2. In the A-498 kidney cancer cell line, shikonin treatment resulted in a significant reduction in protein levels at 48 h, followed by a rebound at 72 h, with expression levels nearing those observed in the untreated A-498 control group. These findings suggest a cell-line-dependent, time-specific modulation of LAMTOR3 in response to shikonin (Figure 10). The results show a partial concordance with the mRNA expression patterns. The observed discrepancies are likely due to cell-line-specific or time-dependent translational regulations, which may contribute to further fine-tuning of protein abundance [23].

Overall, Western blot analyses reveal that the protein expression patterns of the investigated targets during the 24–72 h shikonin treatment period were largely consistent with their corresponding mRNA expression profiles.

### 3.6. Pathway Analysis Showed Interaction of Shikonin Leading to Apoptosis and Renal Disease

IPA confirmed that the target molecules identified in our study (ERK, FOXO1, FOXO3, JNK1, PDCD4, AKT3) form an interactive network with shikonin, which further leads to renal cancer, renal cell carcinoma, and apoptosis (Figure S1). The relationship of each molecule with renal disease and necrosis was also established. The target molecules that contribute to the renal cancer and dysfunction endpoint are known to be specifically associated with apoptosis of mesangial cells, podocytes, and glomerular cells, with necrosis of renal tubules and kidney cell lines. MiR-16-5p, which is closely related to miR-15b, contributes to diseased states along with miR-181a-5p. The molecules in bold were the ones identified in our analysis (Figure S1).

## 4. Discussion

Apoptosis is a form of programmed cell death that is tightly regulated by genetic mechanisms and plays a vital role in maintaining cellular homeostasis. Physiologically, apoptosis occurs in cells that are no longer needed, such as those eliminated during developmental processes. It can also be triggered under pathological conditions, including infections, tumor formation, and irreversible cellular damage, where cells undergo controlled self-destruction for the benefit of the organism [24,25].

As outlined in the introduction, shikonin is a natural naphthoquinone compound derived from *Lithospermum erythrorhizon*, which has demonstrated notable anticancer activity across a variety of tumor cell lines, including human lung adenocarcinoma cells. Its effects are concentration-dependent, influencing both the degree and type of cell death [26]. Earlier studies have also shown that shikonin exhibits dose-dependent cytotoxicity: at lower concentrations, it predominantly induces apoptosis via intrinsic pathways, while at higher concentrations, necrosis becomes more prominent [26,27].

Cancer development is also influenced by alterations in oncogenes and tumor suppressor genes, whose expression can be regulated by miRNAs. These genes and their associated miRNAs are involved in key mechanisms of cancer initiation and progression, including cell proliferation, growth, and apoptosis [28].

Based on the above findings, we aimed to explore the apoptotic responses induced by shikonin in two studied human renal carcinoma cell lines (CAKI-2 and A-498) as well as assess the regulatory role of selected miRNAs (miR-15b, miR-99b and miR-181a) within these processes.

In general, the examined miRNAs showed a characteristic decrease in CAKI-2 cells following 72 h of shikonin treatment, while in A-498 cells their expression was increased. The differences between the two cell lines are likely related to the distinct morphological and genetic features specific to CAKI-2 and A-498 cells.

These findings suggest that miR-15b, miR-99b, and miR-181a may exert their roles in apoptosis through different modulation of their specific targets, depending on the cellular context. The expression patterns of the studied miRNAs suggest that they may act as tumor suppressors or have oncogenic roles in response to shikonin-induced stress [11,16].

According to in silico database analyses for miR-15b, miR-99b, and miR-181a, we identified seven targets—AKT3, PDCD4, FOXO1, FOXO3, JNK1, ERK and LAMTOR3—that might share a common link to the MAPK/ERK apoptotic signaling pathway [29,30,31,32].

Based on the literature, the overexpression of miR-15b may contribute to the development of multidrug resistance in gastric cancer cells, potentially by targeting the apoptotic protein BCL-2, a key regulator of apoptosis [33].

Evidence from prior studies suggests that overexpression of miR-15b counteracts the inhibitory effects of sunitinib on migration and colony formation in ccRCC cell lines. Moreover, in vivo mouse xenograft models exhibited restored tumor growth in sunitinib-treated mice with elevated miR-15b expression [34].

Several studies have also shown that elevated miR-15b can inhibit apoptosis by activating the PI3K/Akt/mTOR signaling pathway while suppressing the JNK pathway. Therefore, it is inferred that downregulation of miR-15b by shikonin may favor JNK1 activation, enhancing apoptotic signaling [35,36].

Considering the existing findings and the experimental findings of our current study, we propose that miR-15b presumably regulates the MAPK/ERK apoptotic pathway primarily in CAKI-2 cells through direct modulation of the investigated targets AKT3, FOXO1, FOXO3, PDCD4, JNK1, LAMTOR3 and ERK. In A-498 cells, shikonin’s effects appear to involve miR-15b; however, apoptosis is primarily triggered by shikonin’s direct action on apoptotic targets.

MiR-181a-5p is a conserved microRNA that regulates pathological processes such as angiogenesis, inflammation, and obesity. MiR-181a has been associated with promoting fibrosis and extracellular matrix accumulation. Its reduction could, therefore, shift the balance toward apoptosis rather than cell survival in cancerous kidney tissue [37].

MiR-181a might act as an oncomiR or tumor suppressor depending on the cancer type, exerting its effects by binding to target mRNAs with partial complementarity, leading to gene suppression [38,39,40].

The observed linear correlation between miR-181a expression and the levels of in silico identified target genes, particularly in A-498 cells under shikonin treatment, suggests a possible indirect or network-level co-regulation rather than direct activation of apoptotic regulators [15].

Prior studies have confirmed that miR-181a directly targets FOXO1 and PDCD4 and that its suppression increases apoptotic sensitivity in various cancers [41,42]. The molecular network confirmed by IPA also showed the key involvement of miR-181a and its association with renal cancer and dysfunction directly or indirectly via JNK1, FOXO1, FOXO3, and PDCD4 (Figure S1).

MiR-99b is a potential tumor suppressor that is often downregulated in cancers, including RCC. Low levels of miR-99b are linked to poor patient survival. Restoration of miR-99b results in inhibition of RCC cell growth and spread and causes cell cycle arrest, partly by targeting the mTOR pathway. This makes it a promising target for RCC diagnosis and therapy [43].

In our study, shikonin downregulated miR-99b in kidney cancer cells, suggesting that it may also regulate apoptosis by upregulating MAPK/ERK pathway targets, as supported by in silico analysis [3].

It is important to note that we also assessed the expression levels of the target proteins FOXO1, FOXO3, PDCD4, AKT3, JNK1 and LAMTOR3 at the protein level using Western blot analysis. Consequently, expression patterns appear to be consistent with those observed at the mRNA level by quantitative real-time PCR.

Following shikonin treatment, an overall continuous increase in the expression of the examined target genes was observed in both CAKI-2 and A-498 throughout the 24–72 h interval, in contrast to the expression pattern of the corresponding miRNAs.

AKT3 is a kinase that is mainly expressed in the brain and some tumors. In renal cancer, it promotes survival and proliferation by inhibiting apoptosis and interacting with the MAPK/ERK pathway. This crosstalk supports tumor growth and contributes to therapy resistance [44]. It is noteworthy that the magnitude and pattern of this upregulation exhibited some cell-line-specific differences. In the CAKI-2 cell line, increased expression was most prominent for AKT3, while in A-498 cells, this protein was decreased. These findings are consistent with recent studies demonstrating differential regulation of AKT3 by shikonin in renal cancer cell lines, highlighting activation of MAPK/ERK and PI3K/Akt apoptotic signaling pathways [44]. These results also prove our earlier studies with shikonin on CAKI-2 and A-498 cell lines [11].

Increased FOXO1 and FOXO3 were also detected in CAKI-2 cells, whereas in the A-498 cells, PDCD4 exhibited the most pronounced mRNA-level elevation. Mammalian forkhead members of the class O (FOXO) transcription factors, including FOXO1 and FOXO3, also have a regulatory role in several biological processes, including stress resistance, metabolism, cell cycle, apoptosis, and DNA repair [45,46]. Based on the literature, these results suggest that in CAKI-2 cells, the pro-apoptotic effects of the studied miRNAs are likely mediated through the regulation of transcription factors associated with the MAPK/ERK signaling pathway, particularly the FOXO proteins [41]. PDCD4 functions as a tumor suppressor and an inhibitor of protein translation and participates in apoptosis regulation. PDCD4 is the binding partner of eIF4A (eukaryotic initiation factor-4A). Binding between the two can lead to the inhibition of eIF4A’s helicase activity to attenuate translation of mRNAs with structured 5′UTR [47,48]. In contrast, in A-498 cells, the observed increase in PDCD4 expression may indicate that shikonin-induced apoptosis may occur independently of changes in transcription factor levels [41].

LAMTOR3 was also among the common targets of the studied miRNAs, miR-15b, and miR-99b. This gene encodes a scaffold protein that functions in the extracellular signal-regulated kinase (ERK) cascade, regulating late endosomal traffic and cell proliferation [29,49]. Consistent upregulation was observed of LAMTOR3 at the mRNA level. Western blot analyses confirmed similarity for the expression pattern of these proteins after 24–72 h’ treatment with shikonin in both of the studied human kidney cancer cell lines.

Our results indicate that the observed elevation of LAMTOR3 may reflect activation of the MAPK/ERK signaling pathway, which plays a crucial role in tumorigenesis, cell proliferation, cell death, and apoptosis regulation [29]. These considerations are also supported by the data presented in our previously published study [11,16].

In other studies, it has also been shown that the activation of the ERK/MAPK signaling pathway can lead to the activation of the transcription factors FOXO1 and FOXO3, which also function as tumor suppressors. As a result, tumor cell activity is reduced, ultimately triggering apoptosis [50,51].

The c-Jun N-terminal kinases (JNKs), part of the MAPK family, include three isoforms (JNK1, JNK2, JNK3). In renal cell carcinoma, the JNK pathway—particularly JNK1—has a dual role, both promoting and suppressing tumor development, affecting tumor growth, the microenvironment, and treatment response [50]. It is important to highlight the changes in JNK1 expression following shikonin treatment, which showed an initial decrease during the 24–72 h interval, followed by an increase at 72 h in both examined cell lines. This dynamic pattern may be related to the dose- and time-dependent inhibitory effect of shikonin on cell proliferation. According to the literature, JNK1 is a branch of the MAPK signaling pathways that can be activated in response to cellular stress, and prolonged activation of JNK1 is known to exert pro-apoptotic effects. Based on our results, it is plausible that the observed late-phase increase in JNK1 contributes to the induction of apoptosis triggered by shikonin [50].

Notably, the change in miR-181a levels during the 24–72 h shikonin treatment positively correlated with the expression of FOXO1, FOXO3, PDCD4, AKT3, JNK1 and LAMTOR3 target mRNAs in A-498 cells. This correlation does not necessarily imply direct regulation but rather suggests a possible network-level co-regulation or indirect relationship between miR-181a and these targets. Therefore, miR-181a may be indirectly associated with shikonin-induced modulation of the MAPK/ERK pathway, potentially influencing the transcription factors FOXO1 and FOXO3 [52,53]. However, correlation analysis between miR-15b, miR-99b, and their target mRNAs revealed an inverse relationship in both studied cell lines, which is consistent with the inhibitory function of miRNAs. Most likely, these miRNAs regulate their targets directly, although indirect effects through other signaling pathways cannot be excluded. It is therefore plausible that miR-15b and miR-99b contribute to shikonin-induced apoptosis in a network-dependent manner, potentially influencing each other’s effects [36,43]. According to the literature, miR-15b targets anti-apoptotic BCL-2, while miR-99b downregulates mTOR and IGF1R [54].

Based on our results and the background literature, we confirm a central mechanism by which shikonin exerts its effects on modulation of MAPK signaling. Specifically, involvement of the MAPK/ERK pathway and simultaneous activation of the JNK pathway can promote the nuclear translocation of transcription factors FOXO1 and FOXO3 [1]. ERK can also influence FOXO1 and FOXO3 transcription factors, which control the expression of several tumor suppressor genes. We hypothesize that among them, FOXOs might play a particularly important role in inhibiting tumor progression, while their suppression has been linked to increased cell transformation, tumor growth, and angiogenesis [51]. Our previous study demonstrated that shikonin activates pERK and pAKT and induces apoptosis in CAKI-2 and A-498 cells, supporting the involvement of the MAPK/ERK pathway in its cytotoxic effects [6,11,20,21,22]. In this study, we further show that shikonin affects the regulation of target genes by the investigated miRNAs. Future functional studies will aim to dissect the ERK/JNK–FOXO/PDCD4 signaling axis in more detail to clarify the whole molecular mechanisms underlying shikonin-induced apoptosis.

Furthermore, the activated FOXOs, as well-established regulators of apoptosis, may lead to cell death through the activation of downstream targets such as PDCD4, a tumor suppressor protein known to facilitate programmed cell death and suppress tumor progression [1,55,56].

It is believed that the interactions among different miRNAs examined in this study contribute to the regulation of the MAPK/ERK-mediated apoptotic pathway through a more complex, possibly indirect, synergistic mechanism [11,38,57,58]. These interactions highlight the complex interplay between FOXO transcription factors, AKT, PDCD4, and MAPK/ERK signaling under shikonin treatment, orchestrated through specific microRNA expression changes. While our data supports correlation-based interactions, further experimental validation is essential to confirm causality. Notably, key pro-apoptotic and anti-proliferative pathways in renal carcinoma cells operate through a multifaceted mechanism involving ROS generation, MAPK signaling shifts, FOXO activation, and microRNA regulation [59].

These findings underscore the potential of shikonin and miRNA-targeting strategies in the development of novel therapeutic approaches for renal cancers [60].

The divergent apoptotic and molecular responses observed between CAKI-2 and A-498 cells likely reflect their intrinsic biological heterogeneity. Such differences may contribute to their distinct sensitivity to shikonin, as previously reported for RCC subtype-dependent drug responses. This could be one of the reasons for the different signaling responses to shikonin treatment. In our study, these phenotypic differences are also evident in the variable expression of the analyzed miRNAs (miR-15b, miR-99b, miR-181a) and their apoptotic targets (PDCD4, AKT3, JNK1), supporting the notion that shikonin’s efficacy is partly influenced by subtype-specific molecular contexts [11,17,18,22].

The present manuscript does not include experimental in vitro investigations of the direct and indirect targets of the studied miRNAs; therefore, in this regard, the manuscript has certain limitations. However, it provides a detailed discussion of the potential interactions and relationships between the relevant miRNAs and their predicted targets. As a future objective, we aim to experimentally validate and thoroughly investigate the associations between these miRNAs and their specific targets using in vitro models. Future work will also focus on functional validation using miRNA mimics and antagomirs to confirm these interactions experimentally.

## 5. Conclusions

Shikonin induces apoptosis in CAKI-2 and A-498 renal cancer cells by modulating the MAPK/ERK signaling pathway and altering microRNA expression. The altered expression of miR-15b, miR-99b, and miR-181a is associated with changes in the levels of pro-apoptotic targets such as FOXO1, FOXO3, and PDCD4. While downregulation of miR-15b and miR-99b is associated with increased levels of these pro-apoptotic targets, miR-181a shows a transient upregulation in A-498 cells at 48–72 h, correlating with mRNA/protein levels. These microRNAs exhibit context-dependent roles as either tumor suppressors or oncogenes. The different responses between the CAKI-2 and A-498 human kidney cancer cell lines highlight the influence of genetic background on shikonin sensitivity. Overall, shikonin exerts its effects through both direct molecular mechanisms and indirect epigenetic regulations. Further studies are required to validate these interactions and explore their therapeutic relevance.

## Data Availability

The data used to support the findings of this study are included within the article.

## Supplementary Materials

The following supporting information can be downloaded at https://www.mdpi.com/article/10.3390/biomedicines13122898/s1, Figure S1: Ingenuity Pathway Analysis (IPA) illustrating the interactive network of shikonin with molecules implicated in renal disease and dysfunction; Figure S2: Correlation of the relative expression levels of miR-15b, miR-99b, and miR-181a to the level of mRNA expression of FOXO1, FOXO3, PDCD4, AKT3, LAMTOR3 and JNK1; Table S1: List of the primer sequences used for qPCR; Table S2: List of the antibodies used for Western blots; Table S3: TaqMan assay-validated miRNAs and their putative apoptotic targets; Table S4: Validation of Putative Target Genes Expression by qRT PCR. The table represents the fold change ± standard deviation, relative to the control group, based on three technical replicates (*n* = 3) from three independent biological replicates (*n* = 3); File S1. Original blot images.

## Abbreviations

The following abbreviations are used in this manuscript:

Ras	Reticular Activating System
ROS	Reactive oxygen species
PI3K	Phosphoinositide 3-kinase
Akt (PKB)	Protein kinase B
MAPK	Mitogen-activated protein kinase
ERK	Extracellular signal-regulated kinase
JAK	Janus kinase
STAT	Signal transducer and activator of transcription
miRNA	MicroRNA
BCL-2	BCL2 apoptosis regulator
SIRT1	Sirtuin 1
MCL1	MCL1 apoptosis regulator, BCL2 family member
MAPK1	Mitogen-activated protein kinase 1
mTOR	Mammalian target of rapamycin
RCC	Renal cell carcinoma
ccRCC	Clear cell renal cell carcinoma
ATCC	American Type Culture Collection
IMDM	Iscove’s Modified Dulbecco’s Medium
FBS	Fetal Bovine Serum
DMSO	Dimethyl sulfoxide
cDNA	Complementary DNA
qRT-PCR	Quantitative real-time polymerase chain reaction
CT	Cycle threshold
RNU6	U6 small nuclear RNA
mRNA	messenger RNA
GAPDH	Glyceraldehyde 3-phosphate dehydrogenase
PBS	Phosphate-buffered saline
SDS-PAGE	Sodium dodecyl sulfate-polyacrylamide gel electrophoresis
PVDF	Polyvinylidene fluoride
HPRT	Hypoxanthine Phosphoribosyltransferase
CI	Confidence interval
Akt3	Serine/threonine kinase 3
LAMTOR3	Late endosomal/lysosomal adaptor, MAPK and MTOR activator 3
DEDD	Death effector domain-containing protein
NIBAN1	Niban apoptosis regulator 1
SOX4	SRY-box transcription factor 4
JNK (MAPK8)	c-Jun N-terminal kinase (Mitogen-activated protein kinase 8)
TNFSF10	TNF superfamily member 10
MAPK9	Mitogen-activated protein kinase 9
FGF12	Fibroblast growth factor 12
BCL2L11	BCL2-like 11
FOXO1	Forkhead box O1
FOXO3	Forkhead box O3
PDCD4	Programmed cell death 4
JNK1 (MAPK8)	c-Jun N-terminal kinase 1 (Mitogen-activated protein kinase 8)
eIF4A	Eukaryotic initiation factor-4A
IPA	Ingenuity Pathway Analysis
IGF1R	Insulin-like growth factor 1 receptor
