# Supplementary material for: Potential Regulatory Role of miR-15b, miR-99b, and miR-181a of the Shikonin-Induced MAPK/ERK Apoptotic Signaling Pathway in Renal Carcinoma
Source: Biomedicines. 2025 Nov 27;13(12):2898. doi: 10.3390/biomedicines13122898 (PMC12730689; doi:10.3390/biomedicines13122898)
Supplement: Supplementary file 1 [file biomedicines-13-02898-s001.zip › biomedicines-3945458-supplementary.pdf]

Supplementary Figures and Tables to the manuscript

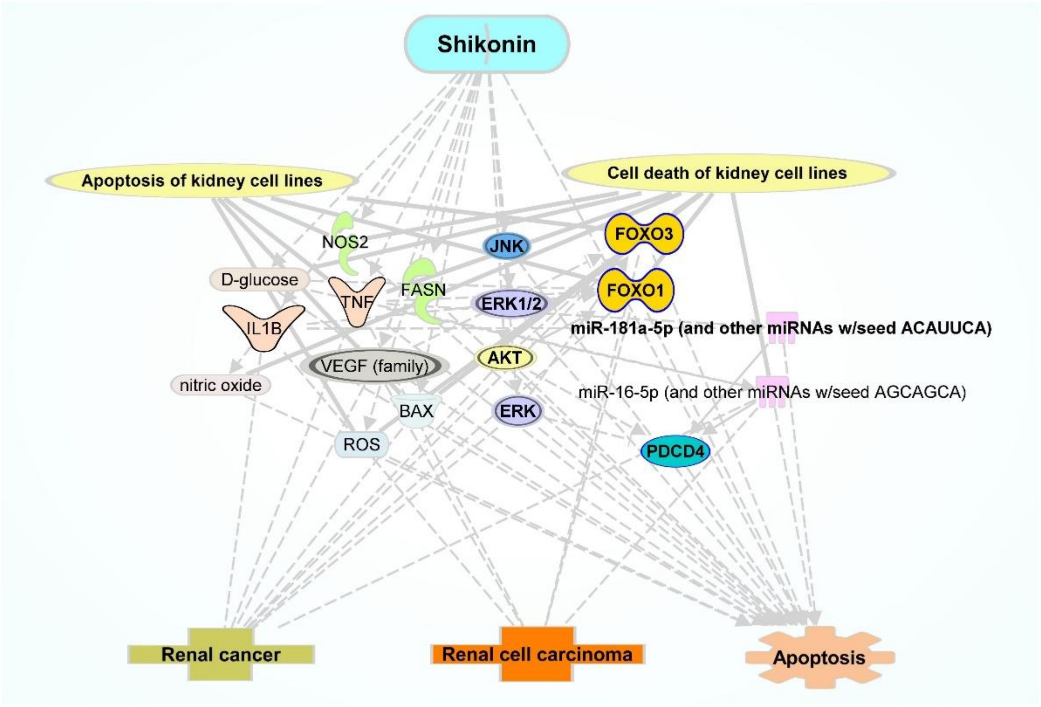

© 2000-2025 QIAGEN. All rights reserved.

Figure S1. Ingenuity Pathway Analysis (IPA) illustrating the interactive network of shikonin with target molecules implicated in renal disease and dysfunction. Solid lines represent direct interactions, while dashed lines indicate indirect interactions. Oval yellow frames highlight the investigated target interaction contributing to apoptosis of kidney cells.

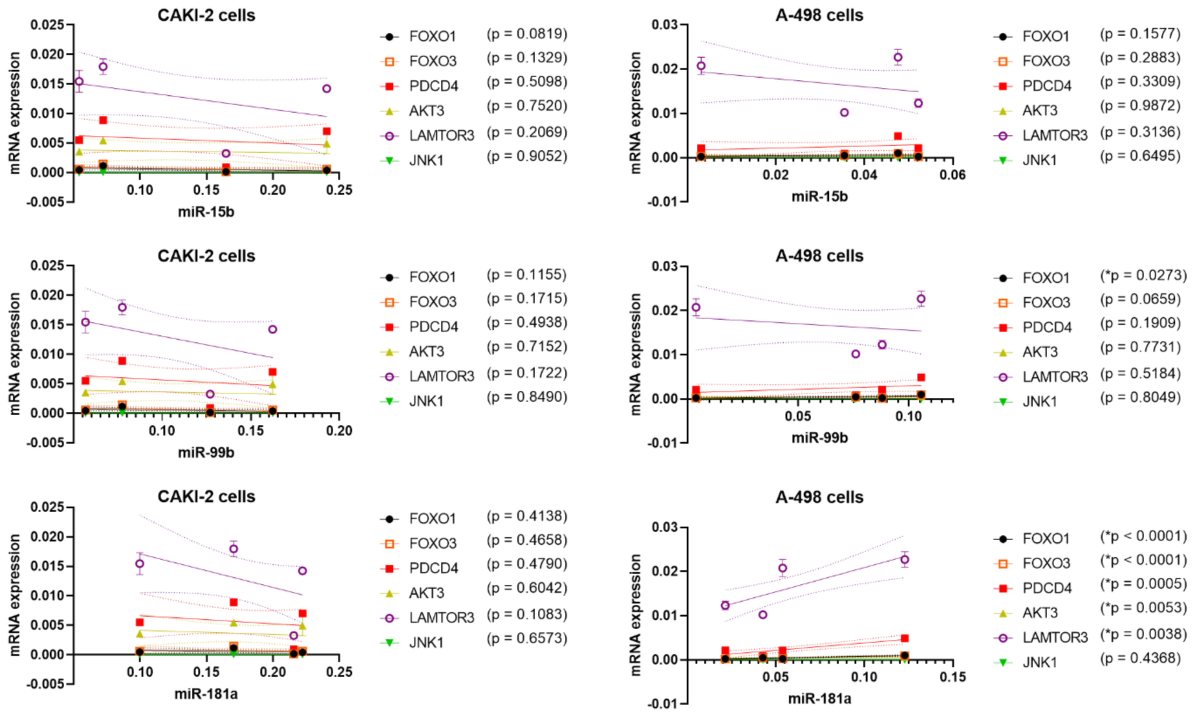

Figure S2. Correlation of the relative expression levels of miR-15b, miR-99b, and miR-181a to the level of mRNA expression of FOXO1, FOXO3, PDCD4, AKT3, LAMTOR3 and JNK1.

Supplementary Table 1

Table S1: List of the primer sequences used for qPCR

Primer	Forward	Reverse	Efficiency %
**FOXO1**	5′-CTA CGA GTG GAT GGT CAA GAG C-3′	5′- CCA GTT CCT TCA TTC TGC ACA CG-3′	102
**FOXO3**	5′-TCT ACG AGT GGA TGG TGC GTT G-3′	5′-CTC TTG CCA GTT CCC TCA TTC TG-3′	98,5
**LAMTOR3**	5′-GTG GTG AGT TTC ATA GCC AGC AG-3′	5′-AGA AAC TTC CAC AAC TTG TCT CAG-3′	110
**JNK1**	5′-GAC GCC TTA TGT AGT GAC TCG C-3′	5′-TCC TGG AAA GAG GAT TTT GTG GC-3′	103
**AKT3**	5′-CGG AAA GAT TGT GTA CCG TGA TC-3′	5′-CTT CAT GGT GGC ATC TGT G-3′	99,6
**PDCD4**	5′-ACT GTG CAA ACC AGT CGA AAG G-3′	5′-CCT CCA CAT CAT ACA CCT GTC C-3′	109

**GAPDH**	5′-TGTAGTTGAGGTCAATGAAGGG-3′	5′-ACATCGCTCAGACACCATG-3′	N/A

Supplementary Table 2

Table S2: List of the antibodies used for Western blots

Antibody	Origin, catalog number	Dilution applied
PDCD4 (D29C6) Rabbit mAb	Cell Signalling, #9535	1:1000
FOXO3 (75D8) Rabbit Mab	Cell Signalling, #2497	1:1000
FOXO1 (C29H4) Rabbit mAb	Cell Signalling, #2880	1:1000
JNK1 (2C6) Mouse mAb	Cell Signalling, #3708	1:2000
Anti- HPRT1 (P00492) Rabbit Mab	BOSTER Biological Technology #M00668	1:1000
LAMTOR3/MAPKSP1 (D38G5) Rabbit mAb(VTSZ:38221900)	Cell Signalling, #8168	1:1000

Supplementary Table 3

Table S3: TaqMan assay-validated miRNAs and their putative apoptotic targets. Potential targets for each miRNA were found by screening three different databases. The targets present in all three databases are highlighted in red in the table.

Database / miRNAs	http://mirdb.org/	http://www.targetscan.org/vert_80/	https://dianalab.e-ce.uth.gr/html/diana/web/index.php?r=tarbasev8/index
	**miRDB**	**TargetScan**	**Tarbase**
**miR-15b-5p**	**AKT3, LAMTOR3,** BAG4, BCL2L2, **BCL2,** MAPK9, MAP3K7, **MAPK8,** AATK, DEDD, PTEN, MMP19	BCL2L2, **AKT3, LAMTOR3, MAPK8,** PDCD1, **BCL2,** LATS2, FOSL1, BAG5, MAP2K3, RET, DEDD, BAG4, AATK, AVEN, FASTK, MAP2K6, TPT1	CCND2, RBL1,CCNE1, RBX1, MDM2, CDC25A, ZMAT3, TNFRSF10B, PIDD1, STAT3, CRKL, FZD6, **BCL2,** BIRC5, IGF1R, VHL, CDH1, MAPK9, CTNNB1, **MAPK8,** MAX, **AKT3,** PIK3CA, **LAMTOR3,** VEGFA, XIAP
**miR-99b-3p**	SPRED1, **DEDD, ERK2,** GSK3B, **NIBAN1, SOX4,** DRAM1, **JNK1, TNFSF10, MAPK9, FGF12,**	MMP8, PTPMT1, **NIBAN1, DEDD, TNFSF10,** BAG5, **ERK2,** TNFRSF17, CASP10, DSTYK, BCL2L13, RERG, **JNK1,** MAPK4, FGF14, **SOX4,** G2E3, TNFRSF19, MAP3K7, PRKCB, MMP16, **FGF12, MAPK9,** PDCD10, AEN, AIFM1, BCL2L15, MMP15,	BCL2L11, CASP2, FGF5,
**miR-181a-5p**	LATS1, **AKT3,** BCLAF1, FOXP1, **FOXO1, FOXO3, BCL2,** BCL2L11, PAWR, TNF, BAG4, **LAMTOR3, PDCD4,** MMP14, TNK1, VHL, MAPK8, MKNK2, FOS	**FOXO3, BCL2,** TNF, BAG4, BCLAF1, **FOXO1,** BCL2L11, **LAMTOR3, PDCD4,** TUSC3, VHL, **AKT3,** MAP3K8, MAPK1, MADD, BMF, SCAI, MMP14, FAF1, PDCD6IP	BMI1, **PDCD4,** MET, tp53, DROSHA, DDX5, **FOXO3,** MAPK3, **AKT3, LAMTOR3,** MAPK1, **BCL2,** IGF1R, PTEN, FOS, APC, **FOXO1,** PIK3R3, XIAP, MDM4, MCL1, DAPK1

Supplementary Table 4

Table S4: Validation of Putative Target Genes Expression by qRT PCR. The table represents the fold change ± standard deviation, relative to the control group, based on three technical replicates (n=3) from three independent biological replicates (N=3).

CAKI-2

	**24 h**		**48 h**		**72 h**	
	**Fold change**	**S.D.**	**Fold change**	**S.D.**	**Fold change**	**S.D.**
AKT3	0.111	0.024	0.777	0.295	1.178	0.315
FOXO3	0.264	0.036	0.991	0.189	2.498	0.338
FOXO1	0.414	0.033	1.185	0.196	2.970	0.443
PDCD4	0.131	0.014	0.785	0.028	1.266	0.109
LAMTOR3	0.229	0.026	1.090	0.163	1.262	0.066
JNK1	2.348	0.839	1.390	0.733	1.468	0.802

A-498

	**24 h**		**48 h**		**72 h**	
	**Fold change**	**S.D.**	**Fold change**	**S.D.**	**Fold change**	**S.D.**
AKT3	1.335	0.445	1.537	0.388	2.355	0.976
FOXO3	0.465	0.185	0.491	0.145	2.012	0.577
FOXO1	0.525	0.044	0.460	0.146	2.167	0.283
PDCD4	2.398	0.142	2.365	0.401	5.575	0.846
LAMTOR3	1.310	0.300	2.050	0.122	2.230	0.233
JNK1	0.952	0.241	0.623	0.141	2.654	0.890
